# Advances in Our Clinical Understanding of Autonomic Regulation Therapy Using Vagal Nerve Stimulation in Patients Living With Heart Failure

**DOI:** 10.3389/fphys.2022.857538

**Published:** 2022-04-21

**Authors:** Marvin A. Konstam, Douglas L. Mann, John James E. Udelson, Jeffrey L. Ardell, Gaetano M. De Ferrari, Martin R. Cowie, Helmut U. Klein, Douglas D. Gregory, Joseph M. Massaro, Imad Libbus, Lorenzo A. DiCarlo, Javed Butler, John D. Parker, John R. Teerlink

**Affiliations:** ^1^ The CardioVascular Center at Tufts Medical Center, Boston, MA, United States; ^2^ Center for Cardiovascular Research, Washington University School of Medicine, Saint Louis, MO, United States; ^3^ UCLA Neurocardiology Program of Excellence, University of California, Los Angeles, Los Angeles, CA, United States; ^4^ Cardiology, Department of Medical Sciences, University of Torino, Turin, Italy; ^5^ School of Cardiovascular Medicine and Sciences, King’s College London, London, United Kingdom; ^6^ Division of Cardiology, University of Rochester Medical Center, Rochester, NY, United States; ^7^ Clinical Cardiovascular Science Foundation, Boston, MA, United States; ^8^ Department of Biostatistics, School of Public Health, Boston University, Boston, MA, United States; ^9^ LivaNova USA Incorporated, Houston, TX, United States; ^10^ Department of Medicine, University of Mississippi Medical Center, Jackson, MS, United States; ^11^ University of Toronto, University Health Network, Toronto, ON, Canada; ^12^ Section of Cardiology, San Francisco Veterans Affairs Medical Center and School of Medicine, University of California, San Francisco, San Francisco, CA, United States

**Keywords:** autonomic nervous system, autonomic regulation therapy, cardiomyopathy, heart failure, left ventricular ejection fraction, neuromodulation, vagus nerve stimulation, guideline directed medical therapy (GDMT)

## Abstract

The ANTHEM-HF, INOVATE-HF, and NECTAR-HF clinical studies of autonomic regulation therapy (ART) using vagus nerve stimulation (VNS) systems have collectively provided dose-ranging information enabling the development of several working hypotheses on how stimulation frequency can be utilized during VNS for tolerability and improving cardiovascular outcomes in patients living with heart failure (HF) and reduced ejection fraction (HFrEF). Changes in heart rate dynamics, comprising reduced heart rate (HR) and increased HR variability, are a biomarker of autonomic nerve system engagement and cardiac control, and appear to be sensitive to VNS that is delivered using a stimulation frequency that is similar to the natural operating frequency of the vagus nerve. Among prior studies, the ANTHEM-HF Pilot Study has provided the clearest evidence of autonomic engagement with VNS that was delivered using a stimulation frequency that was within the operating range of the vagus nerve. Achieving autonomic engagement was accompanied by improvement from baseline in six-minute walk duration (6MWD), health-related quality of life, and left ventricular EF (LVEF), over and above those achieved by concomitant guideline-directed medical therapy (GDMT) administered to counteract harmful neurohormonal activation, with relative freedom from deleterious effects. Autonomic engagement and positive directional changes have persisted over time, and an exploratory analysis suggests that improvement in autonomic tone, symptoms, and physical capacity may be independent of baseline NT-proBNP values. Based upon these encouraging observations, prospective, randomized controlled trials examining the effects on symptoms and cardiac function as well as natural history have been warranted. A multi-national, large-scale, randomized, controlled trial is well underway to determine the outcomes associated with ART using autonomic nervous system engagement as a guide for VNS delivery.

## Introduction

Heart failure (HF) causes a progressive decline in health and physical capacity, and is the leading cause of hospitalization in the elderly (Dharmarajan and Rich, 2017; Kolte et al., 2017). When symptoms of HF persist despite guideline directed medical therapy (GDMT), patients experience progressive fatigue, dyspnea, inability to perform activities of daily living, successive hospitalizations for symptom and co-morbidity management, and ultimately death.

The autonomic nervous system reacts reflexively to HF by stimulating the cardiovascular system in attempt to maintain cardiac output and blood pressure. This reflexive stimulation is maladaptive to a failing heart, however, and ultimately causes HF to progress. While drugs, devices, and cardiac rehabilitation have improved symptoms and survival rates by the progression of HF, HF hospitalization rates and mortality remain high. Although survival from ischemic heart disease has improved, HF prevalence has continued to rise, mainly due to an increase in the prevalence of cardiovascular (CV) risk factors and an increasingly aging population.

In a recently published population-based cohort study of over 300,000 patients, HF was listed on 42% of death certificates and considered to be the cause of 7% of all deaths (Coleman et al., 2011; Brenner et al., 2012; Siegel et al., 2012). The life expectancy of heart failure patients has been less than the survival rates for bowel, breast, and prostate cancer (Coleman et al., 2011; Brenner et al., 2012; Siegel et al., 2012). Survival rates for patients with HF have increased only gradually over the past two decades to 76% at 1 year, 46% at 5 years, and 25% at 10 years (Taylor et al., 2019). About a quarter of patients who are admitted to hospital for HF are readmitted within a month and up to two-thirds within a year, usually for recurrence of HF (Cowie et al., 2014). Individuals who are readmitted with worsening or recurrent symptoms of HF are at a high risk of progressive decline (Neumann et al., 2009).

Existing medical treatment for heart failure and reduced LVEF (HFrEF) using guideline directed medical therapy (GDMT) is directed toward counteracting harmful neurohormonal activation using a combination of beta-blockade, angiotensin-converting enzyme inhibition, angiotensin-receptor blockade in combination with neprilysin inhibition, and mineralocorticoid receptor antagonism.

HF markedly affects patients’ quality of life. Fear, anxiety, and depression are common, and work, travel, social, and leisure activities are affected. Despite the establishment of new prognosis-improving strategies for treatment, patients who continue to have reduced physical capacity, deteriorated quality of life, and hospitalizations for worsening HF desire additional remedies (Jeon et al., 2010; Ågren et al., 2011). The emotional costs are also high for caregivers looking after a member of the family with heart failure (Strömberg, 2013).

In patients with persistent symptoms of advanced HF despite GDMT, electrical stimulation to modulate biological targets within the autonomic nervous system to achieve more physiologic balance between sympathetic and parasympathetic activity has the potential to delay HF progression. Neuromodulation can be achieved with the delivery of autonomic regulation therapy (ART) by stimulating the cervical vagus nerve, which is readily accessible in the neck and provides access to the appropriate central and peripheral nervous system targets for neuromodulation (Salavatian et al., 2017; Hanna et al., 2018).

We will describe the pathophysiology of the autonomic nervous system in heart failure, the progress that has been made in our approach to optimizing VNS delivery for maximal benefit, and a pathway toward clarifying the role of vagus nerve stimulation (VNS) in managing patients with heart failure and reduced left ventricular ejection fraction (LVEF (HFrEF).

## Autonomic Dysregulation Associated With Heart Failure

**FIGURE 1:**
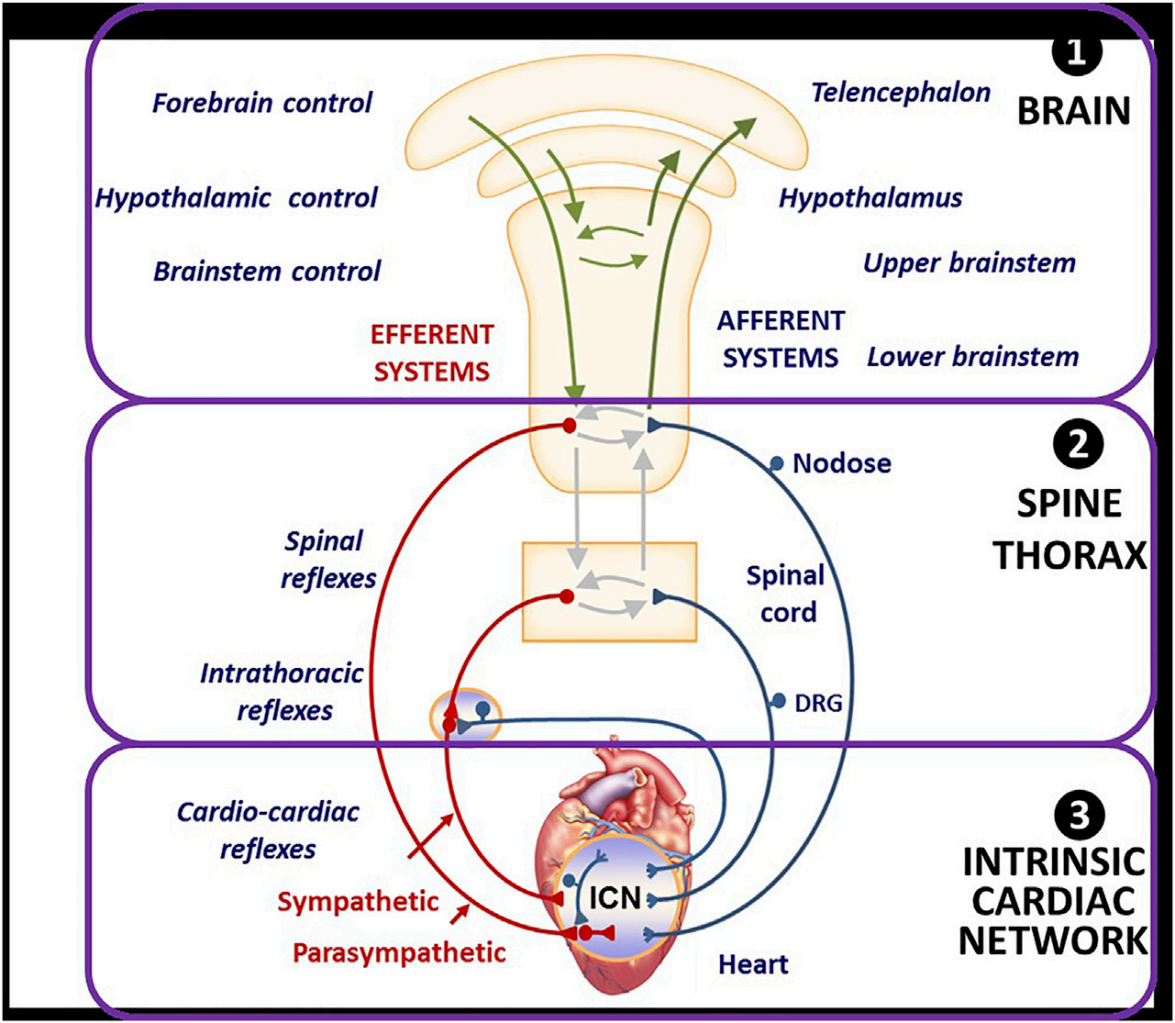
Organization and Function of Autonomic Nervous System for Neuromodulation and Cardiac Control. DRG = Dorsal root ganglia, a group of cell bodies responsible for the transmission of sensory messages from receptors such as thermoreceptors, nociceptors, proprioceptors, and chemoreceptors, to the central nervous system. ICN = intracardiac network, a “little brain” of the heart comprising intracardiac ganglia and interconnecting neurons making adjustments of the cardiac mechanical and electrical activity comprising intracardiac ganglia and interconnecting neurons. From Hanna H, Shivkumar K, and Ardell J. Card Fail Review 2018; 4: 92–98, with permission.

In health, homeostasis is maintained through afferent and efferent sympathetic and parasympathetic interactions that occur throughout the neurocardiac reflex hierarchy comprising the brain, peripheral neural circuits, and the intrinsic cardiac network in the cardiac fat pads (Figure 1). Nervous system activities are integrated within and between the peripheral ganglia and the central nervous system to provide networked control of the heart. At the nerve-myocyte interface, normal parasympathetic activity limits norepinephrine release from sympathetic nerve terminals, engendering cellular resistance to stress (Salavatian et al., 2017; Hanna et al., 2018).

With progressive HF, there is a generalized increase in sympathetic activity and a concurrent decrease in parasympathetic tone throughout the neurocardiac network (Hauptman and Mann, 2011; Kishi, 2012; Florea and Cohn, 2014). The biological consequences of these changes are summarized in Table 1.

**TABLE 1:** Summative biological effects of autonomic nervous system dysregulation.

Loss of Sympathovagal Balance
• Increased sympathetic activation (Henning and Sawmiller, 2001)
• Renin-angiotensin system activation (Tsutsumi et al., 2008)
O_2_ Supply-Demand Mismatch
• Reduced coronary flow (Gamboa et al., 2016)
• Increased oxidative stress (Tracey, 2007)
• Endothelial dysfunction, vasoconstriction (Tracey, 2002)
Inflammation
• Immune system activation and inflammation (Zhang et al., 2009b), (Goldberger et al., 2019)
Myocardial Injury, Fibrosis, and Remodeling
• Apoptotic gene expression, necrosis (Uemura et al., 2007)
• Direct myocardial injury (Mizuno et al., 2010)
• Myocardial remodeling and fibrosis (Gronefeld and Hohnloser, 2003)
Arrhythmias
• Sinus tachycardia (Wu and Vaseghi, 2020)
• Supraventricular tachycardia, atrial fibrillation^56^
• Ventricular tachycardia, ventricular fibrillation^57^

## Administration of Autonomic Regulation Therapy Using Vagus Nerve Stimulation and Cardiovascular Consequences of Restoring Autonomic Balance

There have been more than three decades of clinical experience with VNS to modulate afferent and efferent neurological pathways as an approved therapy for drug-refractory epilepsy and treatment-resistant depression. *In-vitro* and *in vivo* investigations of HF during this time have included explorations of right versus left versus bilateral stimulation, electrode and waveform configuration, efferent versus afferent versus bidirectional stimulation, continuous versus cyclic stimulation, duration of stimulation on/off cycles and number of pulses delivered per cycle, stimulation frequency, open-loop stimulation independent of any trigger, closed-loop stimulation with a physiologic trigger such as ventricular depolarization, current amplitude, effects of VNS intensity on heart rate (HR), and safety and tolerance of VNS titration (De Ferrari and Schwartz, 2011; Ardell et al., 2017). These have provided major contributions to our understanding of the potential use of VNS for the treatment of patients with HFrEF (Supplementary Appendix S1).

Preclinical studies have demonstrated that monotherapy with VNS improves LV ejection fraction, decreases LV end-systolic and end-diastolic volumes, improves indices of LV diastolic function, significantly reduces LV end-diastolic circumferential wall stress, a determinant of myocardial oxygen consumption, and decreases plasma levels of n-terminal pro-brain natriuretic peptide, and reduces of minimum, average and maximum heart rate. These measures suggest that VNS can reduce preload, improve LV relaxation and improve LV function without increasing myocardial oxygen consumption (Premchand et al., 2014; Hanna et al., 2018). Vagal stimulation, performed shortly after the onset of an acute ischemic episode in conscious animals with a healed myocardial infarction, has been effective in preventing ventricular fibrillation (Vanoli et al., 1991). The addition of VNS to beta-blockade improves LV systolic function, improves indices of LV diastolic function, lowers LV end-diastolic pressure and LV end-diastolic wall stress, and increases deceleration time of rapid mitral inflow velocity beyond that seen with beta-blockade alone (Sabbah, 2011).

**FIGURE 2:**
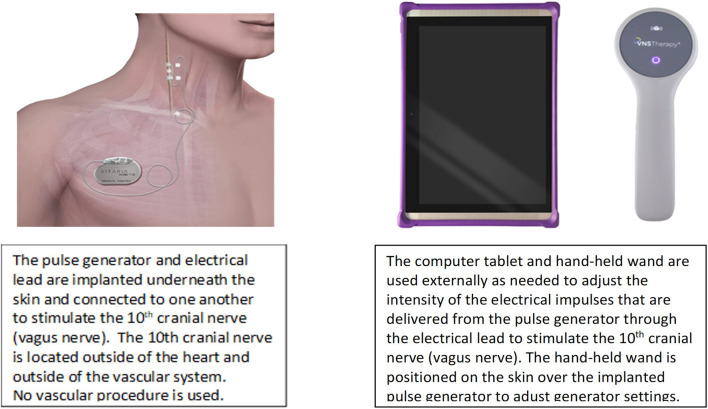
VNS system to deliver ART. Adapted from Anand IS, et al. ESC Heart Fail 2020 with permission.

VNS can be delivered clinically using open-loop stimulation with a programmable pulse generator and an electrode lead that surrounds the cervical vagus nerve and requires no intraoperative mapping for placement (Figure 2) (Inder S et al., 2019).

**FIGURE 3:**
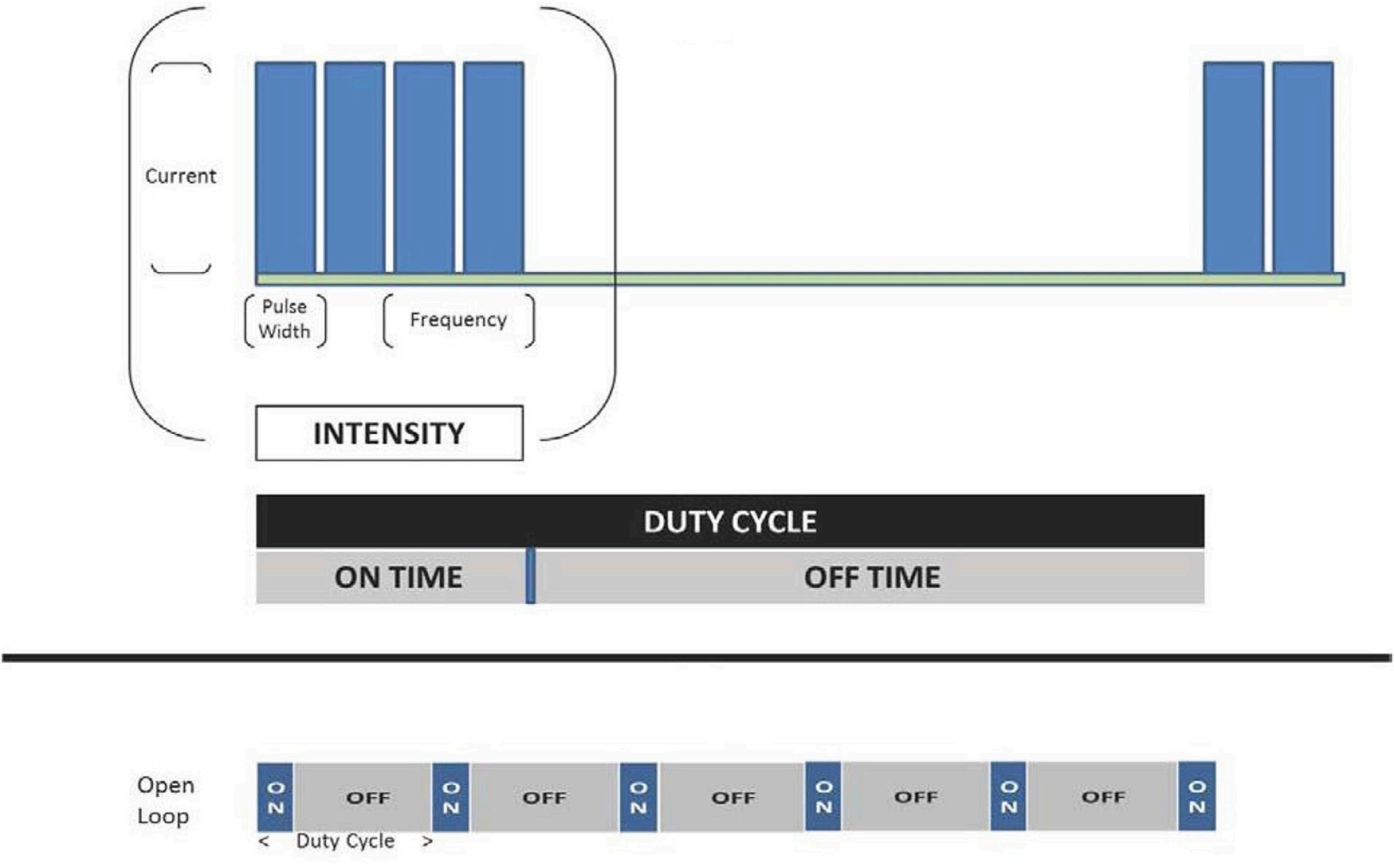
VNS intensity encompasses a combination of pulse amplitude (current), pulse frequency, pulse duration, and duty cycle. Adapted from Anand IS, et al. Int J Neurol Neurother 2019 with permission.

An external programmer, similar to the type used to adjust sensing and stimulation of a pacemaker, can be used to adjust variables of VNS intensity. Intensity comprises the combination of the pulse amplitude (current), pulse frequency, pulse duration (width), and duty cycle. The duty cycle consists of a period of vagus nerve stimulation (“on-time”) alternating with a period of no stimulation (“off-time”), is quantified as the ratio of duration of the on-time to duty cycle duration (i.e., on-time plus off-time), and is repetitive over time (Figure 3).

The electrical lead of the VNS system that is in current investigational use for HF has a bipolar silicone helical design with platinum-iridium conductors, is self-sizing and atraumatic, and is identical in design to the electrical lead that has been utilized in more than 31,000 VNS system implantations for epilepsy or depression and has had 72,100 device-years of patient exposure since 2009 (LivaNova Inc, Houston TX, United States). Cumulative lead survival has exceeded design requirements and there have been a low rate of complications, with common causes being infection (0.87%) and vocal cord dysfunction (0.68%), and a low rate of lead failure. Feasibility studies of chronic utilization of this VNS system for HF have had no device or therapy-related serious adverse events, device malfunctions, or therapy discontinuations (Anand et al., 2020a).

The axons that comprise the cervical vagus nerve include approximately 80% afferent and 20% parasympathetic preganglionic efferent projections (Libbus et al., 2016). The efferent vagal fibers that are directed to the heart usually operate with discharge frequencies in the range of 5–10 Hz (Olshansky et al., 2008). VNS for epilepsy and VNS for heart failure both manifest a dose (intensity)-dependency in achieving the desired therapeutic effect, but differs in the neurologic pathways targeted, the technology platforms utilized, VNS characteristics, and the anatomic portal for VNS administration. Left cervical VNS is used for epilepsy and cathodal stimulation is directed caudally. Right cervical VNS is used for HF and cathodal stimulation is directed peripherally. The therapies also differ in the frequency, pulse width, duty cycle, and current outputs that are utilized. Titration of VNS for epilepsy is empiric while a biomarker of autonomic nervous system engagement and cardiac control is used to guide titration of VNS for HF (Inder S et al., 2019).

The functional biological effects of VNS for HF are achieved through a combination of efferent and afferent stimulation. Efferent effects occur through cathode stimulation in a caudal direction at the interface of the electrical lead with the various axons that traverse the vagus nerve interface. An acute decrease in heart rate detected at the time of stimulation of the vagus nerve during the duty cycle is indicative of autonomic engagement at the time of stimulation (Ardell et al., 2015).

Afferent activation affects efferent sympathetic and parasympathetic function centrally by modulating continuous sympathetic and parasympathetic activity (tonic effect) and activity at rest (basal effect). The actions result peripherally in vasorelaxation through activation of the nitric oxide pathway (Premchand et al., 2014; Hanna et al., 2018). Vagus nerve efferent activation causes anti-adrenergic effects both within the intrinsic cardiac nervous system and via pre-synaptic and post-synaptic interactions at the end terminus (DiCarlo et al., 2013).

At the myocyte level, increases in acetylcholine signaling through muscarinic receptors reduce oxidative stress, decrease contractile force, improve calcium signaling, and restore gene expression. At the same time, cholinergic trans-differentiation of sympathetic neurons occurs, playing a normalizing role against sympathetically mediated pathogenesis (Premchand et al., 2014; Hanna et al., 2018).

**FIGURE 4:**
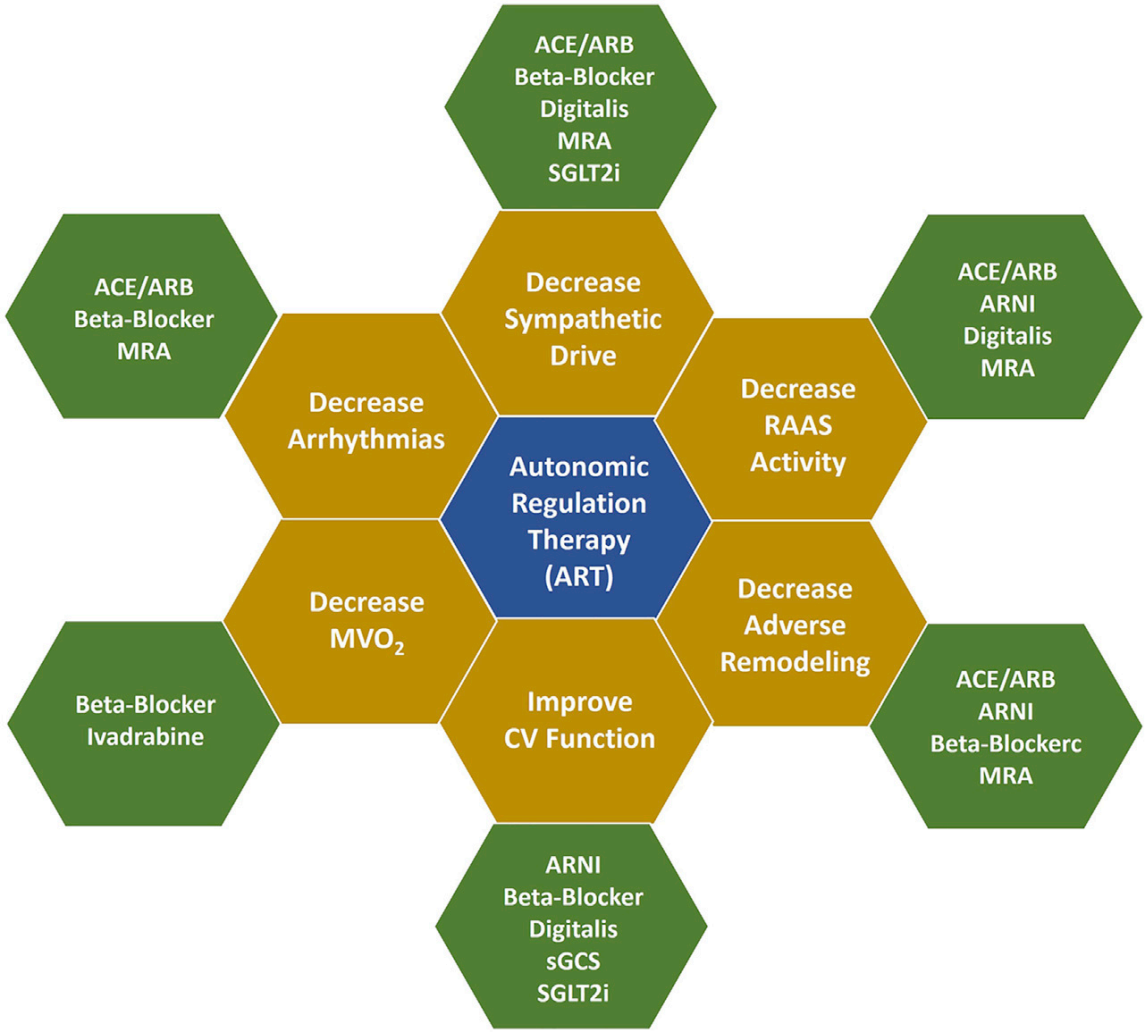
Mechanisms of action of ART and GDMT. Figure adapted from Konstam MA, et al. Circ Heart Fail 2019 with permission. Additional information is provided in Supplementary Appendix S1, S2

As a consequence of augmenting parasympathetic tone and reducing sympathetic hyperactivity, there is a medium-to long-term reduction of endothelial dysfunction, mitigation of cardiac remodeling, improved left ventricular function with restoration of physiologic sympathetic myocyte responsiveness, improved myocardial oxygen supply-demand ratio, and a reduced risk for arrhythmias that compromise cardiovascular function and/or longevity. These effects are complementary to those of pharmacotherapy for HFrEF (Figure 4), (Ardell et al., 2016) and improvements have occurred in symptoms and funcion when VNS is administered in conjunction with GDMT to patients with HFrEF.

## Outcomes of Clinical Studies and Subsequent Analyses of ART using VNS in Patients Living with HFrEF

### Design and Conduct of ANTHEM-HF, INOVATE-HF, and NECTAR-HF Studies of ART using VNS

The designs and the clinical outcomes of the ANTHEM-HF, INOVATE-HF, and NECTAR-HF studies have been published previously (Hauptman et al., 2012; De Ferrari et al., 2014; Zannad et al., 2015; Gold et al., 2016; De Ferrari et al., 2017; Anand et al., 2020b). Each assessed the use of VNS for ART in patients with HFrEF and ongoing symptoms despite GDMT. A comparison of the three study designs is summarized in Table 2. The recommendations for background HF therapy were similar for all three studies. VNS was titrated in all three studies after VNS System implantation.

**TABLE 2:** Similarities and differences among ANTHEM-HF, INOVATE-HF, and NECTAR-HF study designs.

	ANTHEM-HF	INOVATE-HF	NECTAR-HF
Study Phase	2	3	2
Sample Size (N = )	60	707	96
Treatment Assignment	Left vs. Right VNS^a^	Randomized	Randomized
Entry Requirements			
Background Therapy	GDMT	GDMT	GDMT
NYHA Class	2 or 3	3	2 or 3
LVEF (%)	≤35	≤40	≤35
Primary Endpoint	Safety	Death or HFH	LVESD
Secondary Endpoints	MLWHFS, 6MWD, LVEF	KCCQ, 6MWD, NYHA	MLWHFS, NYHA, SFHS
Randomization	1:1^a^	1:2	1:2
Control Arm	---	GDMT	GDMT + Sham VNS
Treatment Arm	GDMT + LVNS GDMT + RVNS	GDMT + RVNS	GDMT + RVNS
VNS System	Open Loop^b^	Closed Loop^c^	Open Loop^b^
Electrical Lead (Cathode Polarity)	Caudal	Cephalad	Caudal
VNS Frequency	10 Hz	1–2 Hz	20 Hz

ANTHEM-HF, AutoNomic Regulation Therapy to Enhance Myocardial Function in Heart Failure. GDMT, Guideline Directed Medical Therapy. INOVATE-HF, increase of Vagal Tone in Heart Failure; LVEF, Left Ventricular Ejection Fraction; KCCQ, Kansas City Cardiomyopathy Questionnaire; LVESD, Left ventricular end-systolic diameter; LVNS, Left Cervical Vagus Nerve Stimulation; MLWHFS, Minnesota Living with Heart Failure Score; NECTAR-HF, Neural Cardiac Therapy for Heart Failure. N = number. NYHA, New York Heart Association; RVNS, Right Cervical Vagus Nerve Stimulation; SFHS, Short Form Health Survey. vs. = versus.

a Randomization to GDMT + RVNS or GDMT + LVNS. No Control Arm of GDMT alone.

b Timing of stimulation is independent of any external signal such as ventricular depolarization (no intracardiac lead is required for system operation).

c Timing of stimulation is linked to the occurrence of ventricular depolarization (implantation of an intracardiac lead is required for system operation). From Inand AS, et al; ESC Heart Fail 2020; 7: 76–84, with permission.

### Differences in Utilization of VNS for ART and Comparative Outcomes

In ANTHEM-HF, VNS was delivered at 10 Hz to mimic the natural operating frequency of the vagus nerve. VNS titration was well-tolerated and completed with confirmation of autonomic engagement using changes in heart rate (HR) dynamics that occurred during the on-time of the duty cycle as a biomarker. Evidence of this engagement was associated with long term improvements from baseline in mean HR and mean HR variability (SDNN) as measured using 24 h ambulatory ECG monitoring, LVEF, 6-min walk duration (6MWD), Minnesota Living with Heart failure (MLWHF) questionnaire mean score, and NYHA (Table 3)(Gold et al., 2016).

**TABLE 3:** ANTHEM-HF Study results.

	Baseline	6 Months	*p*-value
HR (24 Hr)	78 ± 12	72 ± 10	<0.005
HRV (SDNN, ms)	95 ± 29	106 ± 43	<0.01
LVEF (%)	33 ± 7	38 ± 10	0.0001
6MWD (m)	288 ± 64	348 ± 77	<0.0001
QoL (MLWHF)	39 ± 12	20 ± 9	<0.0001
NYHA (I/II/III/IV)	0/26/20/0	26/21/2/0	<0.0001

SDNN, Standard Deviation of Normal-to-Normal RR intervals. *p*-values based on paired t-tests at 6 months. From Premchand RK et al. J Cardiac Fail 2016; 22: 639–42, with permission.

In INOVATE-HF, VNS administered at a lower frequency of 1–2 Hz was not associated with any long-term improvements in mean HR or mean HR variability. The primary efficacy endpoint of a composite of death or HF hospitalization showed no significant between-group difference, though improvements occurred in the secondary endpoint outcomes of NYHA class, KCCQ mean score, and 6-min walk distance (De Ferrari et al., 2014).

VNS titration in NECTAR-HF could not be completed in the majority of patients due to side effects (e.g., neck pain and coughing) that occurred with VNS even at low current amplitudes when using a stimulation frequency of 20 Hz. There were no significant long-term improvements in mean HR or mean HR variability. At 6 months of follow up, VNS did not reduce the primary efficacy endpoint, LV end-systolic diameter, or other secondary echocardiographic measures. There were significant improvements in the secondary outcomes of MLWHF mean score and NYHA (Anand et al., 2020b). A subsequent analysis showed by means of a new technique that subtle HR changes were detectable during VNS at 20 Hz in only 13/106 (12%) of ambulatory ECG recordings obtained at 6 and 12 months. The absence of this response was at variance with prior results in animal studies with the same device (Howland, 2014; Anand et al., 2020b).

**FIGURE 5:**
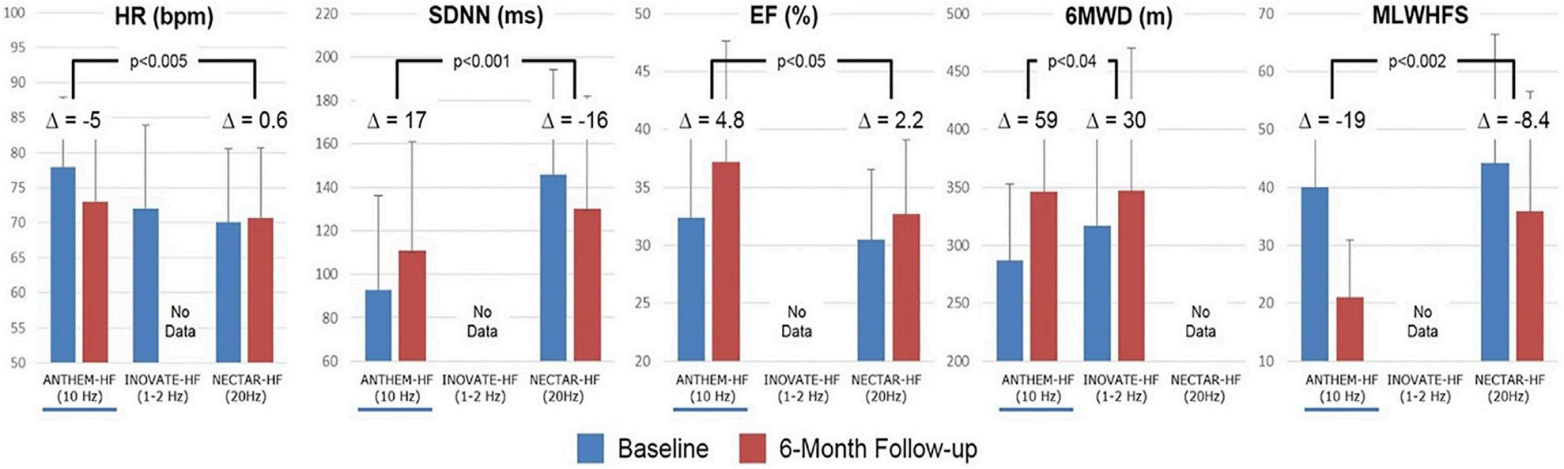
Stimulation frequencies used for VNS and associated changes from baseline in symptomatic and functional outcomes in the treatment arms of ANTHEM-HF, INOVATE-HF, and NECTAR-HF. △ = difference; % = percent; bpm = beats per minute; Hz = Hertz; m = meters; ms = milliseconds; other abbreviations as in the text. *p*-values are two-sided and are based on two-sample t-tests. From Inand AS, et al. ESC Heart Fail 2020; 7: 76–84, with permission.

Post-hoc comparisons of the stimulation frequencies that were used for VNS, and the associated symptomatic and functional outcomes that were observed in the treatment arms in these three studies have been published and are summarized in Figure 5. In ANTHEM-HF, a small but statistically significant change in HR dynamics, indicative of autonomic nervous system engagement, was consistently observed during the on-times of the VNS duty cycle, and a significant long-term improvement from baseline occurred in mean HR and mean HR variability during 24-h ambulatory ECG recording. These were accompanied by greater and statistically significant long-term improvements from baseline in LVEF, 6MWED, and MLWHF mean score in ANTHEM-HF when compared to the changes that were reported in the other two studies, where either a lower or a higher stimulation frequency was used (Premchand et al., 2019).

There was no difference in efficacy, tolerability, and safety in patients who were randomized to either right VNS system implantation or left VNS system implantation in the ANTHEM-HF study. Comparable efficacy, tolerability, and safety using bilateral cervical VNS has not been studied (Dede et al., 2021).

### Effects of ART Using VNS in Conjunction With GDMT

The background treatment with GDMT in ANTHEM-HF compared favorably with the background therapy administered during two contemporary pivotal studies that supported the approval of new categories of HF therapy (Table 4). (Premchand et al., 2016) For entry into the ANTHEM-HF study, patients were required to have maximally tolerated and stable doses of beta-blocker therapy administered for at least 3 months and all other indicated classes of oral pharmacologic therapy for HFrEF administered for at least one month (Gold et al., 2016).

**TABLE 4:** Comparison of background pharmacologic therapy administered in ANTHEM-HF, PARADIGM-HF, and SHIFT.

	ANTHEM-HF	PARADIGM-HF	SHIFT
N	60	8,442	6,398
Minimum duration of stable GDMT (months)	3	1	1
ACEi or ARB (%)	85	100^a^	91
β-blockers (%)	100^b^	93	89
β-blocker Dose (% patients administered ≥100% of target/≥50% of target)	26/56	NR	26/56
Loop Diuretic (%)	88	80	83
Mineralocorticoid (%)	75^c^	55	61

NR = not reported. PARADIGM-HF, Efficacy and Safety of LCZ696 Compared to enalapril on Morbidity and Mortality of Patients With Chronic Heart Failure. SHIFT, Ivabradine and Outcomes in Chronic Heart Failure.

a *p*<0.001 vs. ANTHEM-HF and SHIFT

b *p* = 0.031 vs. SHIFT and *p* = 0.002 versus PARADIGM-HF

c *p* = 0.002 vs. PARADIGM-HF and *p* = 0.03 versus SHIFT [Chi-Square analyses]. From Premchand RK, et al; ESC Heart Fail. 2019; 6: 1,052–1,056, with permission.

### Effects of ART using VNS in Conjunction with Beta-Blockade

Similar and statistically significant improvements in symptoms and function with ART were observed in a post-hoc analysis of patients categorized by the percentage of target beta-blocker dose administered at baseline. One hundred % of participating patients received a beta-blocker in dose that was either ≥50% of the maximum target (16 patients, 27%; group 1) or less than 50% of the maximum target (44 patients, 73%; group 2). Heart rate, systolic blood pressure, LVEF, use of ACE/ARB, and use of MRA at baseline were similar for the two groups at baseline. Six months after VNS titration, VNS reduced HR and significantly improved HR variability (SDNN), LVEF, 6MWD, and MLWHFS, compared with baseline, equally in both groups (Sharma et al., 2021).

### Enduring Autonomic Nervous System Engagement and Long-Term CV Effects of ART Using VNS

Long term effects of administering ART using VNS are summarized elsewhere in this issue of the journal. Evaluation of 15 patients with HFrEF after 4.7 ± 0.3 years (range: 4.0–5.0 years) of VNS in the ANTHEM-HF study showed that the majority manifested long term engagement of the autonomic nervous system without apparent deleterious effects of ART upon patient safety, symptoms, or function.

Persistent autonomic nervous system response to long term VNS provides physiologic underpinning for the sustained symptomatic and functional effects observed in association with chronic VNS in larger subsets of the ANTHEM-HF patient population. For patients with available data, statistically significant improvements from baseline in symptoms and function have been reported at 12 months (46 [77%] of the overall sample) and at 42 months (33 [55%]). At 12 months, there were persistent improvements from baseline in HR variability, LVEF, 6MWD, NYHA class, and MLWHFS (Table 5)(Cleland et al., 2009).

**TABLE 5:** Efficacy measures at 12 months in cohort study of ANTHEM-HF patient population (n = 46).

	Baseline	6 Mo	12 Mo	*p-*value
LVEF (%)	33 ± 7	38 ± 10	39 ± 10	<0.0005
6MWD (m)	288 ± 64	348 ± 77	352 ± 62	<0.0005
MLHFQ score	39 ± 12	20 ± 9	18 ± 9	<0.0005
NYHA class (I/II/III/IV)	0/26/20/0	26/21/2/0	32/14/0/0	<0.0005

Abbreviations as in text. *p*-values are two-sided and are based on paired t-tests for 12 month comparison with baseline. From Premchand RK et al. J Card Fail 2016; 22: 639–42, with permission.

At 42 months, there have been no device-related SAEs and no device malfunctions, and there have been statistically significant improvements from baseline in HR variability, LVEF, 6MWD, MLWHFS, and NYHA Class (Table 6). Understandably, generalization of these observations is limited since they are derived from a small subset of the original ANTHEM-HF population comprising a surviving cohort that were willing and able to be followed and a sample size too small to reach definitive conclusions (Zile et al., 2020).

**TABLE 6:** Efficacy measures at 42 months in cohort study of ANTHEM-HF patient population (n = 33).

	Baseline	12 Months	24 Months	30 Months	42 Months	*p-*value
HRV (SDNN, ms)	96 ± 27	107 ± 32	112 ± 44	110 ± 30	107 ± 28	<0.025
LVEF (%)	35 ± 6.9	43 ± 10	42 ± 10	45 ± 12	41 ± 12	<0.005
6MWD (m)	297 ± 62	354 ± 58	359 ± 47	367 ± 40	389 ± 70	<0.0001
MLHFQ score	38 ± 12	17 ± 9	21 ± 11	17 ± 9	10 ± 12	<0.0001
NYHA (I/II/III/IV)	0/19/14/0	23/10/0/0	21/11/1/0	20/12/1/0	20/12/1/0	<0.0001

Abbreviations as in text. *p*-values are two-sided and are based on paired t-tests at 42 months. From Sharma K et al. Int J Cardiol 2021; 323: 175–78, with permission.

A favorable long-term safety profile has also been reported in 91 patients receiving VNS at 18 months in the NECTAR-HF Study, whose first phase was a 6-month randomized controlled evaluation of patients receiving active VNS versus an implanted but inactive VNS system, and second phase was a safety evaluation after activation of the VNS system in all patients at 6 months (Howland, 2014).

### Relationship of Baseline NT-proBNP to Cardiovascular Improvement With ART

Recent heart failure studies have associated lower baseline natriuretic peptide levels with improved morbidity/mortality outcomes during pharmaceutical treatment for HFrEF, (Konstam et al., 2019) and better clinical outcomes during neuromodulation with carotid nerve plexus stimulation for HFrEF when NT-proBNP is less than 1,600 pg/ml (Zhang et al., 2009a). An analysis of variance and logistic regression (for improvement in NYHA) model has been used in an exploratory evaluation of the relationship of baseline NT-proBNP values above and below 1,600 pg/ml to the symptomatic and functional changes that occurred from baseline during VNS in the ANTHEM-HF Pilot Study (Table 7).

**TABLE 7:** Repeated measures analysis of changes associated with VNS in the ANTHEM-HF Pilot Study and relationship to baseline NT-proBNP value.

	Baseline^a^	6 months	Change^b^	*p* ^c^	Regression coefficient^d^	*p* ^e^
HR	78 (10) [n = 60]	73 (11) [n = 57]	−4 (10)	0.0210	1.414 (−2.974, 5.802)	0.528
SDNN	92 (31) [n = 60]	111 (50) [n = 54]	17 (40)	0.0176	1.128 (−19.95, 22.206)	0.916
LVEF	32 (7) [n = 60]	37 (10) [n = 56]	5 (8)	0.0042	−6.547 (−10.60, −2.491)	0.002
6MWD	287 (66) [n = 60]	346 (78) [n = 57]	59 (85)	<0.0001	−25.64 (−58.24, 6.954)	0.123
MLWHFS	40 (14) [n = 60]	21 (10) [n = 57]	−18 (13)	<0.0001	0.881 (−3.569, 5.332)	0.698
NYHA^d^	0/33/24/0 [n = 57]	30/24/3/0 [n = 57]	77%^f^	<0.001	−0.387 (−1.142, 0.367)	0.314

a Mean (± standard deviation)

b Mean (± standard deviation) except NYHA

c *p*-values are two-sided and are based on paired t-tests at 6 months

d The regression coefficients (95% confidence interval) were calculated for continuous variables using the mean outcome for a baseline BNP value ≥1,600 pg/ml minus the mean outcome for a baseline BNP value <1,600 pg/ml; the odds ratio for NYHA is the odds of NYHA improvement for a baseline BNP value ≥1,600 pg/ml versus the odds of NYHA improvement for a baseline BNP value <1,600 pg/ml

e *p*-values are two-sided based on repeated measures generalized-estimating analysis of variance or logistic regression with a baseline NT-proBNP value <1,600 pg/ml or a baseline NT-proBNP value ≥1,600 pg/ml as the independent variable

f 77% of patients improved at 6 months. From Anand IS, et al. Int J Cardiol Heart Vasc.2020; 30: 29:100520, with permission.

The median NT-proBNP value at baseline was 868 pg/ml (Q1-Q3 322 -1875 pg/ml). There was a net 16% decrease from baseline in median NT-proBNP value that during VNS that was modest and non-significant in the study cohort (N = 60).

While LVEF improved overall, there was a statistical interaction between baseline NT-proBNP value above or below 1,600 pg/ml and better LVEF improvement during VNS. Other symptomatic and functional improvement during chronic VNS was independent of baseline NTproBNP value above or below 1,600 pg/ml.

These are preliminary and hypothesis-generating findings, and the ongoing ANTHEM-HFrEF Pivotal Study of VNS may provide additional insights.

## Furthering Our Understanding of ART for Patients Living With HFrEF

A large-scale, multi-national randomized controlled trial (ANTHEM-HFrEF) is now underway in order to fully test the hypotheses raised by results of ANTHEM-HF, and to further assess the impact of ART on morbidity and mortality. The key inclusion criteria are stable GDMT for at least 4 weeks, NYHA class III or class II if hospitalized for HF within the previous 12 months, LVEF ≤35%, LV end diastolic diameter <8.0 cm, NT-proBNP ≥800 pg/ml, and 6MWD of 150–450 m, limited by HF symptoms.

A Bayesian adaptive sample size selection is being utilized in determining the impact of VNS on morbidity and mortality. The effects of ART on symptoms and function are also being evaluated. Patients are randomized 2:1 to VNS plus GDMT or GDMT alone. Evaluation of morbidity and mortality utilizes a conventional frequentist approach for analysis of the primary outcome end point—reduction in the composite of cardiovascular death or first HF hospitalization.

**FIGURE 6:**
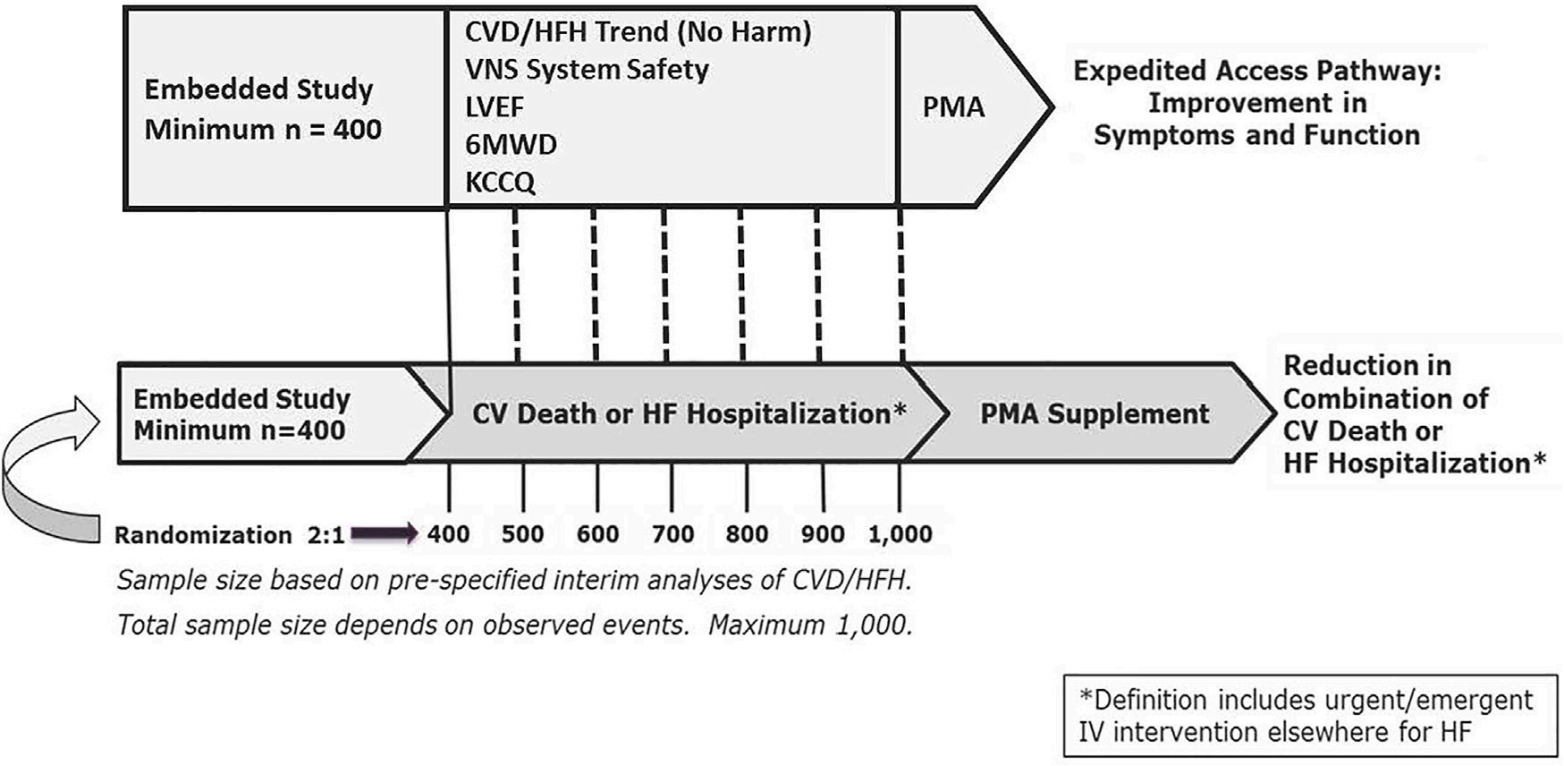
ANTHEM-HFrEF Pivotal Study Design. CVD = Cardiovascular death. HFH = Heart failure hospitalization. Mo = months. N = number. PMA = Pre-Market Application. sPMA = supplementary Pre- Market Application. Dotted lines represent serial interim analyses as appropriate. Other abbreviations as in text. From Konstam MA, et al. Circ Heart Fail 2019; 12: e005879, with permission.

A second study is embedded within the ANTHEM-HFrEF Pivotal Study to evaluate improvement in symptoms and function in the study population. Successful demonstration of improvement in symptoms and function will require meeting two risk-related conditions—a 60% or greater probability of a reduction in the combination of cardiovascular death/HF hospitalization at the end of the study (i.e., no evidence of harm) and sufficient freedom from device-related serious adverse events—and demonstrating statistically significant improvements in three efficacy endpoints: LVEF, 6MWD, and KCCQ overall score (Figure 6) (Zaeem et al., 2019).

## Conclusion

Progress has continued during the past decade in our understanding of how ART may be delivered using VNS in a safe and tolerable manner to alleviate symptoms, increase functional capacity, and improve the quality of life in patients with HFrEF. Findings to date, derived from pilot investigation of VNS delivered at the natural operating frequency of the vagus nerve, have provided hypotheses that are now being tested in a large multicenter randomized investigation of VNS effects on symptoms, functional status, quality of life, morbidity and mortality.
