# Supplementary material for: Advances in Our Clinical Understanding of Autonomic Regulation Therapy Using Vagal Nerve Stimulation in Patients Living With Heart Failure
Source: Front Physiol. 2022 Apr 21;13:857538. doi: 10.3389/fphys.2022.857538 (PMC9068946; doi:10.3389/fphys.2022.857538)
Supplement: Supplementary file 1 [file DataSheet2.docx]

Appendix 2. Modes of action and physiological effects of pharmacotherapies of GDMT for HFrEF.

| Pharmacological Class | Mode of Action and Physiological Effects |
| --- | --- |
| ACE inhibitor | Inhibition of angiotensin-converting enzyme that converts angiotensin I to angiotensin II enhances natriuresis, lowers blood pressure, and prevents remodeling of smooth muscle and cardiac myocytes;^^[[1]](#endnote-1)^^ reduction of sympathetic drive and renin–angiotensin–aldosterone system (RAAS) activity;^^[[2]](#endnote-2)^,^[[3]](#endnote-3)^^ regression of left ventricular remodeling;^^[[4]](#endnote-4)^^ reduction of arrhythmias^^[[5]](#endnote-5)^^ |
| ARB | Inhibition of Angiotensin (AT) II binding to the AT_1_ receptor, enhancing vasodilation and decreasing aldosterone release by the adrenal zona glomerulosa, sodium and water reabsorption in the proximal tubular cells, and vasopressin secretion;^^[[6]](#endnote-6)^^ reduction of sympathetic drive;^^[[7]](#endnote-7)^^ reduction of arrhythmias^^[[8]](#endnote-8)^^ |
| ARNI | Neprilysin inhibition and AT1 receptor blockade, increasing anti natriuretic peptide, plasma and urinary cyclic guanosine monophosphate, renin concentration and activity and angiotensin II levels, inducing natriuresis, diuresis, and vasodilation, inhibition of the RAAS system and the sympathetic nervous system, and beneficial antifibrotic, antiproliferative and antithrombotic effects ;^^[[9]](#endnote-9)^^ regression of left ventricular remodeling and improvement in left ventricular function^^[[10]](#endnote-10)^,^[[11]](#endnote-11)^^ |
| Beta-blocker | Inhibition of chronic beta-1 stimulation-induced myocardial apoptosis/necrosis/inflammation;^^[[12]](#endnote-12)^^ reduction of sympathetic drive;^^[[13]](#endnote-13)^^ regression of left ventricular remodeling, improvement in left ventricular function, and reduction of MVO_2_;^65,^[[14]](#endnote-14)^,^[[15]](#endnote-15)^^ reductio of arrhythmias^^[[16]](#endnote-16)^^ |
| Digoxin | Reduction of sympathetic drive and RAAS activity^^[[17]](#endnote-17)^,^[[18]](#endnote-18)^^ |
| I_F_ inhibitor | Inhibition of the cardiac pacemaker current (If), a mixed sodium-potassium inward current that controls the spontaneous diastolic depolarization in the sinoatrial node and hence regulates the heart rate, with no effect on blood pressure, intracardiac conduction, myocardial contractility, or ventricular repolarization;^^[[19]](#endnote-19)^^ reduction of myocardial oxygen consumption^^[[20]](#endnote-20)^^ |
| MRA | Modulation of renin–angiotensin–aldosterone system, and at the tissue level, limit of potassium and magnesium loss and reduction of myocardial fibrosis and hypertrophy;^^[[21]](#endnote-21)^^ reduction of sympathetic overdrive and RAAS activity;^^[[22]](#endnote-22)^,^[[23]](#endnote-23)^^ regression of left ventricular remodeling and improvement of left ventricular function;^83^ decrease in arrhythmias^83^ |
| sGCS | Stimulates soluble guanylate cyclase, improving endothelial function, vasotone regulation, and decreasing the vascular and ventricular stiffening, fibrosis, and hypertrophy that causes in a decline of heart as well as kidney function;^^[[24]](#endnote-24)^^ improvement of left ventricular function^^[[25]](#endnote-25)^^ |
| SGLT2i | Increases mitochondrial calcium levels and reduces cytoplasmic sodium and calcium levels by inhibiting NHE, and downregulating the activity of Na+/H+ exchanger 3 in the proximal renal tubule, through leading to natriuresis and reduction of heart failure,^^[[26]](#endnote-26)^^reduction of sympathetic drive,^^[[27]](#endnote-27)^^ improvement of left ventricular function^^[[28]](#endnote-28)^^ |

1. Herman LL, Padala SA, Ahmed I, et al. Angiotensin Converting Enzyme Inhibitors (ACEI) [Updated 2021 Dec 26]. In: StatPearls. Treasure Island (FL): StatPearls Publishing; 2022 Jan. <https://www.ncbi.nlm.nih.gov/books/NBK431051/> [↑](#endnote-ref-1)
2. Grassi G, Cattaneo BM, Seravalle G, Lanfranchi A, Pozzi M, Morganti A, Carugo S, Mancia G. Effects of chronic ACE inhibition on sympathetic nerve traffic and baroreflex control of circulation in heart failure. Circulation 1997; 96: 1173-9. doi: 10.1161/01.cir.96.4.1173 [↑](#endnote-ref-2)
3. Ma TK, Kam KK, Yan BP, Lam YY. Renin-angiotensin-aldosterone system blockade for cardiovascular diseases: current status. Br J Pharmacol 2010; 160:1273-92. doi: 10.1111/j.1476-5381.2010.00750.x [↑](#endnote-ref-3)
4. Khattar RS, Senior R, Soman P, van der Does R, Lahiri A. Regression of left ventricular remodeling in chronic heart failure: Comparative and combined effects of captopril and carvedilol. Am Heart J 2001; 142: 704-13. doi: 10.1067/mhj.2001.116768 [↑](#endnote-ref-4)
5. Schupp T; Behnes M; Weiss C; Nienabe, C; Lang S; Reiser L.; Bollow A; Taton G; Reichelt .; Ellguth D; et al. Beta-Blockers and ACE Inhibitors are associated with improved survival secondary to ventricular tachyarrhythmia. Cardiovasc Drugs Ther 2018; 32: 353–363 [↑](#endnote-ref-5)
6. Schmieder RE. Mechanisms for the clinical benefits of angiotensin II receptor blockers. Am J Hypertens 2004; 185: 720–730. doi.org/10.1016/j.amjhyper.2004.11.032 [↑](#endnote-ref-6)
7. Ishikawa S, Takahisa N, Matsunaga K, Mantani K, Takabatake W, Ishizawa M, et al. Effect of Angiotensin II receptor blocker on cardiac autonomic activity in patients with chronic heart failure. J Card Fail 2105; 21: S172. DOI:https://doi.org/10.1016/j.cardfail.2015.08.158 [↑](#endnote-ref-7)
8. Schupp T, Bejnes M, Abumayyaleh M, Weidner K, Mashayekhi K, Bertsch T, Akin I. Angiotensin Converting Enzyme Inhibitors versus Receptor Blockers in patients with ventricular tachyarrhythmias. J Clin Med 2022;

   11: 1460. https://doi.org/10.3390/jcm11051460 [↑](#endnote-ref-8)
9. Tamargo J. The mechanism of action of LCZ696. Card Fail Rev 2016; 2: 40–6. doi.org/10.15420/cfr.2016:1:1 [↑](#endnote-ref-9)
10. Abboud A, Januzzi JL. Reverse Cardiac Remodeling and ARNI Therapy. Curr Heart Fail Rep 2021; 18: 71-83. doi: 10.1007/s11897-021-00501-6 [↑](#endnote-ref-10)
11. Poglajen G, Anžič-Drofenik A, Zemljič G, Frljak S, Cerar A, Okrajšek R, Šebeštjen M, Vrtovec B. Long-Term Effects of Angiotensin Receptor-Neprilysin Inhibitors on Myocardial Function in Chronic Heart Failure Patients with Reduced Ejection Fraction. Diagnostics (Basel) 2020; 10:522. doi: 10.3390/diagnostics10080522 [↑](#endnote-ref-11)
12. Brown NJ and Vaughan DE. Angiotensin-converting enzyme inhibitors. Circulation 1998; 97: 1411–1420. doi.org/10.1161/01.CIR.97.14.1411 [↑](#endnote-ref-12)
13. Malfatto G, Facchini M, Branzi G, Riva B, Sala L, Perego GB. Long-term treatment with the beta-blocker carvedilol restores autonomic tone and responsiveness in patients with moderate heart failure. J Cardiovasc Pharmacol 2003; 42: 125-31. doi: 10.1097/00005344-200307000-00019 [↑](#endnote-ref-13)
14. van Campen LC, Visser FC, Visser CA. Ejection fraction improvement by beta-blocker treatment in patients with heart failure: an analysis of studies published in the literature. J Cardiovasc Pharmacol 1998; 32: S31-5. doi: 10.1097/00005344-199800003-00006 [↑](#endnote-ref-14)
15. Eichhorn EJ, Bedotto JB, Crag RM et al. Effect of -adrenergic blockade on myocardial function and energetics in congestive heart failure. Circulation 1990 ; 82: 473–83. doi: 10.1161/01.cir.82.2.473 [↑](#endnote-ref-15)
16. Lechat P, Hulot J-S, Escolano S. et al. Heart rate and cardiac rhythm relationships with bisoprolol benefit in chronic heart failure in CIBIS II Trial. Circulation 2001; 103: 1428-1433. doi.org/10.1161/01.CIR.103.10.1428 [↑](#endnote-ref-16)
17. Newton GE, Tong JH, Schofield AM, Baines AD, Floras JS, Parker JD. Digoxin reduces cardiac sympathetic activity in severe congestive heart failure. J Am Coll Cardiol 1996; 28:155-61. doi: 10.1016/0735-1097(96)00120-9 [↑](#endnote-ref-17)
18. Covit AB, Schaer GL, Sealey JE, Laragh JH, Cody RJ. Suppression of the renin-angiotensin system by intravenous digoxin in chronic congestive heart failure. Am J Med 1983; 75: 445-7. doi: 10.1016/0002-9343(83)90346-7 [↑](#endnote-ref-18)
19. Tse S, Mazzola N. Ivabradine (Corlanor) for heart failure: The first selective and specific I f Inhibitor. P T 2015; 40: 810-814. PMID: 26681903 [↑](#endnote-ref-19)
20. Colin P, Ghaleh B, Monnet X, Hittinger L, Berdeaux A. Effect of graded heart rate reduction with ivabradine on myocardial oxygen consumption and diastolic time in exercising dogs. J Pharmacol Exp Ther 2004; 308: 236-40. doi: 10.1124/jpet.103.059717 [↑](#endnote-ref-20)
21. Vizzardi E, Regazzoni V, Caretta G, Gavazzoni M, Sciatti E, Bonadei I, Trichaki E, et al. Mineralocorticoid receptor antagonist in heart failure: Past, present and future perspectives. Int J Cardiol Heart Vessel 2014; 3: 6-14. doi: 10.1016/j.ijchv.2014.03.005 [↑](#endnote-ref-21)
22. Vizzardi E, Regazzoni V, Caretta G, Gavazzoni M, Sciatti E, Bonadei I, Trichaki E, Raddino R, Metra M. Mineralocorticoid receptor antagonist in heart failure: Past, present and future perspectives. Int J Cardiol Heart Vessel 2014; 3: 6-14. doi: 10.1016/j.ijchv.2014.03.005 [↑](#endnote-ref-22)
23. Keidar S, Gamliel-Lazarovich A, Kaplan M, Pavlotzky E, Hamoud S, Hayek T, et al. Mineralocorticoid receptor blocker increases angiotensin-converting enzyme 2 activity in congestive heart failure patients. Circ Res 2005; 97: 946-953. doi.org/10.1161/01.RES.0000187500.24964.7A [↑](#endnote-ref-23)
24. Breitenstein S, Roessig L, Sandner P, Lewis KS. Novel sGC Stimulators and sGC Activators for the Treatment of Heart Failure. Handb Exp Pharmacol 2017; 243: 225-247. doi: 10.1007/164_2016_100 [↑](#endnote-ref-24)
25. Lombardi CM, Cimino G, Pagnesi M, Dell'Aquila A, Tomasoni D, Ravera A, et al. Vericiguat for Heart Failure with Reduced Ejection Fraction. Curr Cardiol Rep 2021;2 3:144. doi: 10.1007/s11886-021-01580-6 [↑](#endnote-ref-25)
26. Lam CSP, Chandramouli C, Ahooja V, Verma S. SGLT-2 Inhibitors in Heart Failure: Current Management, Unmet Needs, and Therapeutic Prospects. J Am Heart Assoc. 2019 Oct 15;8(20):e013389. doi: 10.1161/JAHA.119.013389. Epub 2019 Oct 12. PMID: 31607208 [↑](#endnote-ref-26)
27. Herat LY, Magno AL, Rudnicka C, Hricova J, Carnagarin R, Ward NC, Arcambal A, Kiuchi MG, Head GA, Schlaich MP, Matthews VB. SGLT2 Inhibitor-Induced Sympathoinhibition: A Novel Mechanism for Cardiorenal Protection. JACC Basic Transl Sci 2020; 5:169-179. doi: 10.1016/j.jacbts.2019.11.007 [↑](#endnote-ref-27)
28. Santos-Gallego CG, Vargas-Delgado AP, Requena-Ibanez JA, Garcia-Ropero A, Mancini D, Pinney S, et al for the EMPA-TROPISM (ATRU-4) Investigators. Randomized Trial of Empagliflozin in Nondiabetic Patients With Heart Failure and Reduced Ejection Fraction. J Am Coll Cardiol 2021; 77: 243-255. doi: 10.1016/j.jacc.2020.11.008 [↑](#endnote-ref-28)
