# Supplementary material for: Advances in Our Clinical Understanding of Autonomic Regulation Therapy Using Vagal Nerve Stimulation in Patients Living With Heart Failure
Source: Front Physiol. 2022 Apr 21;13:857538. doi: 10.3389/fphys.2022.857538 (PMC9068946; doi:10.3389/fphys.2022.857538)
Supplement: Supplementary file 2 [file DataSheet1.docx]

Appendix 1.

**Anatomy and Physiology of the Vagus Nerve, Physiological Effects of VNS, and Mode of Action of ART**

The axons that comprise the cervical vagus nerve include approximately 80% afferent and 20% parasympathetic preganglionic efferent projections.^^[[1]](#endnote-1)^^ The efferent vagal fibers that are directed to the heart usually operate with discharge frequencies in the range of 5 to 10 Hz.^^[[2]](#endnote-2)^^

As described by Hadaya et al, the mode of action of ART is based upon the physiological effects of VNS on the cardiac neuraxis (Figure 7) that are mediated through direct efferent activation of the vagus nerve, resulting in activation of parasympathetic postganglionic cells that are located in the intrinsic cardiac nervous system (ICNS) and act on muscarinic receptors on the myocardium. Local circuit neruons (LCNs) within the ICN are activated, which modulate sympathetic reflexes with input from afferent soma, ultimately blunting reflexes within the ICNS. Afferent activation results in reduced central drive and modulates projections to spinal sympathetic networks. Sympathetic spinal reflexes are also blunted via inhibitory projections (not shown).^58^


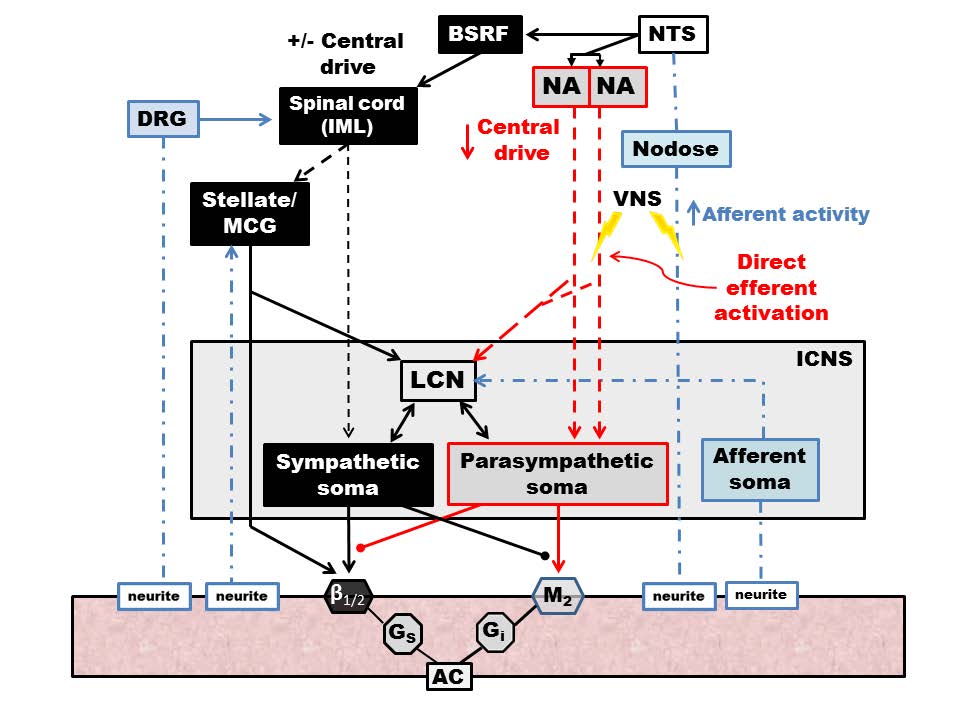
Figure 7. Mode of action of ART. From Hadaya J and Ardell JL. Front Physiol 2016 with permission.^58^ Abbreviations: NTS, nucleus tractus solitarus. NA, nucleus ambiguus. BSRF, brain stem reticular formation. VNS, vagal nerve stimulation. DRG, dorsal root ganglia. IML, intermediolateral nucleus. MCG, middle cervical ganglia. LCN, local circuit neuron. ICNS, intrinsic cardiac nervous system. AC, adenylyl cyclase.

Preclinical studies of the contribution of vagal afferents on efferent control of cardiac function have evaluated right and left cervical VNS sequentially in the intact state, following ipsilateral or contralateral vagus nerve transection, and following bilateral transection. VNS utilized currents from 0.25-4.0 mA, frequencies from 2-30 Hz, and a 500 μs pulse width. Right or left VNS evoked significantly greater current- and frequency-dependent suppression of chronotropic, inotropic and lusitropic function subsequent to sequential transection. The thresholds for afferent-mediated effects on cardiac parameters were 0.62 ± 0.04 mA and 0.65 ± 0.06 mA with right and left VNS. Afferent-mediated tachycardias were maintained following beta-blockade. The findings demonstrate that vagal afferents inhibit centrally-mediated parasympathetic efferent outflow and the ipsilateral and contralateral vagus nerves exert a substantial buffering capacity that is exerted at multiple levels of the cardiac neural hierarchy.^^[[3]](#endnote-3)^^

The acute effects of VNS on cardiac control have been quantified using electrocardiographic RR-interval dynamics in 60 patients with HFrEF and randomized to either right VNS system implantation or left VNS system implantation. VNS was titrated using a pulse width of 250 microseconds, a pulse frequency of 10 Hz, and a target output current amplitude of 1.5–3.0 mA using a VNS duty cycle comprising a 14-second period of VNS administration (on-time) followed by a 66-seconds when VNS is no longer administered (off-time). A decease in HR when VNS administered was used to confirm engagement of the autonomic nervous system engagement during VNS (Figure 8). This decrease in HR correlated to VNS current amplitude, pulse width, and frequency. HR decreased more in patients with right VNS (-2.22 ± 0.13 bpm) than in the patients with left VNS (-0.60 ± 0.08 bpm, P < 0.001). There was stronger linear correlation between stimulus intensity and lengthening of the R-R interval among patients with right-sided VNS implantation (r = 0.88, P < 0.0001) than among the 31 patients with left-sided VNS implantation (r = 0.49, P < 0.002).^^[[4]](#endnote-4)^^


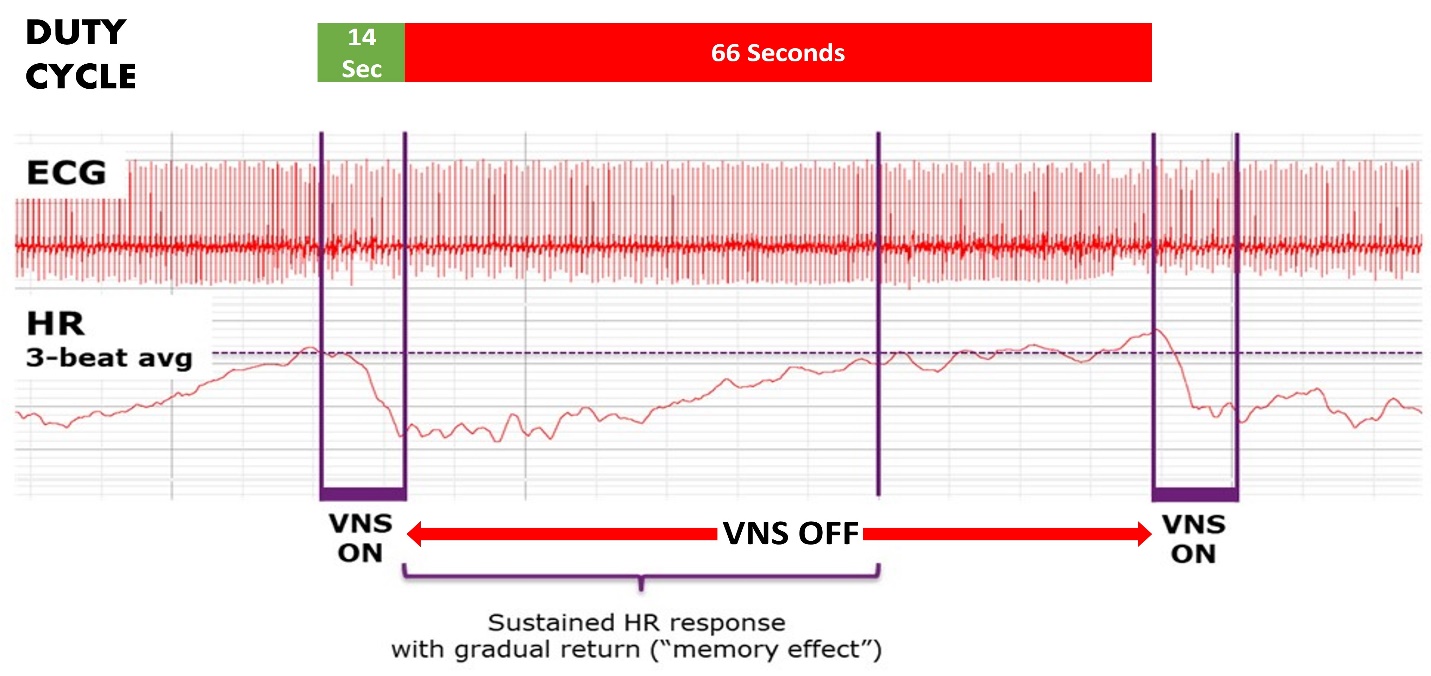


Figure 8. Transient HR decrease when VNS is administered (ON) during the VNS duty cycle. The response persists with a gradual return to baseline during the period when VNS is no longer administered (OFF) during the VNS duty cycle. VNS parameters: 2.0 mA, 10 Hz, and 250 µs with duty cycle 14 sec ON / 66 sec OFF. Patient recording during ANTHEM-HF Study (on file).^30^

1. Hadaya J, Ardell JL. Autonomic Modulation for Cardiovascular Disease. Front Physiol 2020; 11: 617459. doi: 10.3389/fphys.2020.617459 [↑](#endnote-ref-1)
2. Qing KY, Wasilczuk KM, Ward MP, Phillips EH, Vlachos PP, Goergen CJ, Irazoqui PP. B fibers are the best predictors of cardiac activity during Vagus nerve stimulation. Bioelectron Med 2018; 4: 5. doi: 10.1186/s42234-018-0005-8 [↑](#endnote-ref-2)
3. Ardell JL, Rajendran PS, Nier HA, KenKnight BH, Armour JA. Central-peripheral neural network interactions evoked by vagus nerve stimulation: functional consequences on control of cardiac function. Am J Physiol Heart Circ Physiol 2015; 309: H1740-1752 doi.org/10.1152/ajpheart.00557.20150 [↑](#endnote-ref-3)
4. Nearing BD, Libbus I, Amurthur B, Kenknight BH, Verrier RL. Acute Autonomic Engagement Assessed by Heart Rate Dynamics During Vagus Nerve Stimulation in Patients With Heart Failure in the ANTHEM-HF Trial. J Cardiovasc Electrophysiol 2016; 27: 1072-7. doi: 10.1111/jce.13017 [↑](#endnote-ref-4)
